# FLT3 inhibitor design using molecular docking based virtual screening for acute myeloid leukemia

**DOI:** 10.6026/97320630015104

**Published:** 2019-02-28

**Authors:** Padmini Gokhale, Aashish Pratap Singh Chauhan, Anushka Arora, Natasha Khandekar, Anuraj Nayarisseri, Sanjeev Kumar Singh

**Affiliations:** 1 In silico Research Laboratory,Eminent Biosciences,Mahalakshmi Nagar,Indore-452010,Madhya Pradesh,India.; 2 Bioinformatics Research Laboratory,LeGene Biosciences Pvt Ltd.,Mahalakshmi Nagar,Indore-452010,Madhya Pradesh,India; 3 Computer Aided Drug Designing and Molecular Modeling Lab,Department of Bioinformatics,Alagappa University,Karaikudi-630 003,Tamil Nadu,India

**Keywords:** FLT3 Inhibitors, Acute Myeloid Leukemia, Molecular Docking, Virtual Screening

## Abstract

Acute Myeloid Leukaemia (AML) is a blood cancer, which affects the red blood cells in the bone marrow. Of the possible proteins that are affected in AML, fms-like tyrosine kinase 3 (FLT3) has long been recognized as a potential therapeutic target as it affects the other signaling pathways and leads to a cascade of events. First-generation inhibitors sorafenib and midostaurin, as well as secondgeneration agents such as quizartinib and crenolanib are known. It is of interest to identify new compounds against FLT3 with improved activity using molecular docking and virtual screening. Molecular docking of existing inhibitors selected a top scoring bestestablished candidate Quizartinib having PubChem CID: 24889392. Similarity searching resulted in compound XGIQBUNWFCCMASUHFFFAOYSA-NPubChemCID: 44598530 which shows higher affinity scores. A comparative study of both the compounds using a drug-drug comparison, ADMET studies, boiled egg plot and pharmacophore parameters and properties confirmed the result and predicted the ligand to be an efficient inhibitor of FLT3.

## Background

The incidence frequency of AML increases with age, from 1.3 per 100,000 in a population of patients who are less than 65 years old. And, 12.2 cases per 100000 in a population of patients over 65 years. Considering the current treatments, as much as 70% of patients are 65 years or older and have lower survival rates, which are mostly confirmed from 1 year after diagnosis. AML has a considerable tendency to occur in children due to genetic factors. It is slightly more prevalent in men than women. In the United States for the year 2019, about 61,780 new cases of leukemia and 22,840 leukemia-related deaths have occurred. An increase to about 21,450 new cases of acute myeloid leukemia (AML) mostly in adults and 10,920 deaths from AML again almost all are adults are obtained from the population consensus. The five-year overall survival rate for acute myeloid leukemia is only 27.40 percent.

The patho-physiology of Acute Myeloid Leukemia is such that it arises in patients with an underlying hematological disorder, or as a serious side effect of prior therapy, for example, exposure to topo-isomerases II, alkylating agents or radiation being the key factors. But, moreover, we see that in most of the cases it appears as a de novo or new malignancy in previously healthy individuals [1]. The pathogenesis of AML involves the abnormal proliferation and differentiation of a clonal population of myeloid stem cells. It progresses rapidly, with myeloid cells interfering with the production of normal white blood cells, red blood cells, and platelets. This also results in the accumulation of poorly differentiated myeloid cells.

addition to large chromosomal rearrangements, molecular changes have also added to the development and prognosis of AML. We see that genetic mutations are identified in more than 97% of cases, often in the absence of any large chromosomal abnormality. There are 3 classes of mutations in acute myeloid leukemia. [2] The class I mutations result in the activation of proproliferative pathways. The class II mutations impair normal hematopoietic differentiation in order for leukemia to develop. These 2 classes occur in conjunction with each other. Common class I mutations, such as FLT3 (internal tandem duplications, ITD, and tyrosine kinase domain mutations, TKD), K/NRAS, TP53, and c-KIT are found in 28, 12, 8 and 4% of cases, respectively [3]. Mutations in receptor tyrosine kinases, for example, FLT3 duplications are seen up to 50% of AML cases and statistically denote a worse prognosis. Alterations in genes involved in epigenetic regulation have recently emerged as a third class of mutations, with downstream effects on both cellular differentiation and proliferation that is, the class I and class II type mutations [4]. The identification of recurrent genetic mutations is considered in FLT3. It’s prognostic impact of FLT3- ITD complex interaction may depend on the presence of biallelic mutations [5]. FLT3 tyrosine kinase receptor is located on chromosome 13 (13q12) of the human genome.

Fatigue, anorexia and weight loss, bleeding, bruising, infection, red spots on the skin, or shortness of breath are symptoms of acute myeloid leukemia. If AML is left untreated, it will lead to immediate death within months of diagnosis. Young adults have undergone multicenter treatment trials [6] and the survival rates are calculated [7]. Acute Myeloid Leukemia has 6 major classifications namely, AML with recurrent genetic abnormalities, AML with myelodysplasia-related changes, therapy-related myeloid neoplasms, myeloid sarcoma, myeloid proliferations related to Down syndrome and AML not otherwise specified. These can be broken down to have 11 subtypes collectively.

## Methodology

### Selection of FLT3 inhibitors:

Literature findings were conducted to find out the pre-established inhibitors of FLT3 for acute myeloid leukemia ( Table 1). These inhibitors were selectively sorted out on the basis of its binding capacity to the protein, in turn deciding the behavior of the complex formed. The total numbers of inhibitors selected for further analysis were 47. 3D Structures of these inhibitors were obtained. Most of these structures are available in the PubChem database from where it was directly downloaded. Some of the unavailable structures were built using molecular editor software, MarvinSketch. All of them were saved in 3D.sdf format.

### Protein and ligand Preparation:

The crystal structure of the target protein was obtained from the Protein Data Bank with PDB ID: 4RT7 [37]. In a similar way, compiling all the 3D structures of ligands using the LigPrep module of Schrodinger suite, 2013 (Schrodinger did the ligand preparation. LLC, New York, NY) where, these were optimized through OPLS 2005 force field algorithm [38-42]. Both the structure files of the protein and the ligands were saved for the ease of processing for docking analysis. All the ligand structures were saved under a single file using a .sdf extension [43-47].

### Molecular Docking:

Molecular docking analysis was carried out using Molegro Virtual Docker (MVD), which unifies high potential Piece-Wise Linear Potential (PLP) and MolDock scoring function [48-54]. The preexisting ligand was removed from the complex structure of the FLT3 protein structure and all its cavities were prepared using Molegro Virtual Docker [55-57]. The cavity one, having the largest volume was selected, verifiably the one in which the previous ligand was docked. The preferred orientation of one molecule with the other to form a stable complex was found after docking the .sdf file of the set of ligands with the target protein. The docking procedure holding parameters were a maximum iteration of 1500, grid solution 0.2 having a binding affinity and a maximum population size 50. The protein and ligands were assessed on the subsequent conformation of the Internal Electrostatic interaction (Internal ES), sp2-sp2 torsions, and internal hydrogen bond interaction [58-62]. Post-docking, energy minimization and H-bond optimization were carried out. Placing of Simplex Evolution at max steps 300 and neighbor distance faster 1.00. This confirms that the predominant molecule obtained after docking forms a stable complex. After docking to minimize the complex energy of ligandreceptor interaction the Nelder Mead Simplex Minimization (using non- grid force field and H-bond directionality) was used [63-66].

### Virtual Screening:

Molecular docking provided us with the best-established drug for the target protein. This drug was predominantly selected due to its high negative re-rank score, which tells us about its high binding affinity. A similarity search was performed to obtain a superior compound from the whole database, apart from the already established compounds. This database known as the PubChem database is developed by NIH is one of the public chemical repositories containing structures of 93 million chemical compounds. The filtration property parameter set by component rule of Lipinski's rule of five was set at threshold >=95 [67-69]. These set of potential hits of compounds were downloaded in .sdf format and docked with the crystal structure of FLT3 protein. The superiority of the virtual screened compound is tested with the preestablished drug in the next step.

### Drug-drug comparative study:

The completion of the docking process leads to the formation of a docking file. The first docking file contained all the information of the established compound and the protein cavity [38-39]. This particular file was opened with the help of Molegro Virtual docker software. All the constraints, cavities and ligands in the structurecomplex were removed to obtain only the protein structure [70-73]. The best pose of the drug was tallied from the result generated and was then imported. The resultant structure generated was saved as the best-posed drug and was stored in PDB format, for that particular compound [74-76]. These steps were repeated for the second docking file containing all the information of the virtual screened compound and the protein cavity. The best fit was studied and an excel sheet was organized to check and compare all the affinities, hydrogen interaction, steric energy and high re-rank score to draw out a comparison between the two drugs.

### ADMET studies:

The admetSAR database is an open and free interface, which takes into account the absorption, distribution, metabolism, elimination, and toxicity (ADMET) of the drug molecules [38-39]. These tell us about the pH, solubility and the overall physicochemical properties of drugs to study the drug metabolism and drug-drug and drug-body interactions. AdmetSAR database is available at http://lmmd.ecust.edu.cn:8000/ for analysis of a particular drug. The properties and parameters help inproviding us with essential information related to the development and discovery of drugs. The admetSAR database mostly consists of 22 qualitative classifications and 5 quantitative regression models, which gives us a high precision based predicted outcome. Hence, the estimation of the properties of the compounds was done using admetSAR. The best docked established compound Quizartinib having PubChem CID- 24889392 and the best virtual screened compound with PubChem CID-44598530were considered and the bioactivity properties and toxicity were predicted by using admetSAR [77-79].

### Softwares, Suites and Web servers Used:

The 3D chemical structures were retrieved from NCBI's PubChem database in 3DSDF format. Some compounds that lack PubChem ID or the 3D structure was unavailable in PubChem were drawn with the help of MarvinSketch5.6.0.2, (1998-2011, ©ChemAxonLtd). Schrodinger suite was used for the optimization of ligands (Schrodinger, LLC, 2009, New York, NY). The flexible docking was achieved by taking receptor protein structure and all ligand compounds in Molegro Virtual Docker 2010.4.0.0. Molecular Visualization was done with the assistance of Accelrys Discovery Studio® Visualizer 3.5.0.12158 (Copyright© 2005-12, Accelrys Software Inc.). ADMET profiles were obtained and tabulated using admetSAR (Laboratory of Molecular Modeling and Design© 2012 East China University of Science and Technology, Shanghai Key Laboratory for New Drug-Drug Design).

### Boiled-egg plot:

The Brain Or IntestinaL EstimateD permeation method (Boiled Egg) is an accurate predictive model that works by computing the lipophilicity and polarity of small molecules. It is a graphical classification model, which provides readouts on the basis of two parameters - human gastrointestinal absorption (HIA) and Blood Brain Barrier (BBB). The regions of the Egg plot can be easily studied on the basis of yellow-colored yolk representing the physiochemical space for highly probable BBB crossing drug and the white space representing the physiochemical space for HIA absorption. Yolk and white areas are not mutually exclusive. The readouts are based on two physiochemical descriptors, WLOGP and TPSA, which tells us of the lipophilicity and apparent polarity of the compound [38-39]. Another parameter, involved in this study is the P-gp active efflux pump as it transports the lipophilic drug out of the brain capillary endothelial cells that form the BBB [38-39]. The drug to be studied is also classified as the P-gp substrate (PGP+; blue dots) and P-gpnonsubstrate (PGP-; red dots), which provide the absorption and distribution of the drug in the central nervous system (CNS). Thus, we get to know the distribution rate and localized accumulation of the drug for passive absorption (inside/outside the white), passive brain access (inside/outside the yellow) and active efflux from CNS to the gastrointestinal tract (GI). In order to get the functional properties of the drug for target protein FLT3, the mapping of the parameters of GI and BBB and optimization of the BOILED-Egg plot was done by using the rerank affinity of the docking results of the pre-established drug and the virtual screened compound.

## Results and Discussion:

### Docking results:

The docking results of the pre-established 65 drugs, established Quizartinib as the compound showing best interaction and best minimum re-rank score (Table 2). This compound has PubChem CID-24889392 and shows the highest affinity score directed towards our target protein. Its properties include a molecular weight of 560.673 g/mol, hydrogen bond donor count of 2 and hydrogen bond acceptor count of 8. The logPvalue is established at 5.6. Hence, this compound has a greater inhibitory effect on the protein FLT3.

### Virtual Screening Results:

Virtual screening of the best-established compound against PubChem database ensued in a total number of 109 compound structures that showed a similarity percentage of =>95. Table 3 enlists top 10 compounds that display the highest affinity to the target protein based on their re-rank scores. The compound with PubChem CID 44598530 was observed to have the lowest re-rank score, which made it the best virtual screened compound. Physical properties of this compound include a molecular weight of 651.801 g/mol, a hydrogen bond donor count of 2, a hydrogen bond acceptor count of 10 and a logP value of 5.4. The re-rank score of this compound stands at -190.091 and the H-bond interaction score at -5.93428.

### Drug-drug comparative study:

Table 4 provides a comparative account of value and re-rank scores of the best-established drug and the best virtual screened drug. It brings about the estimate between the similarities and dissimilarities of the 2 best drugs when they are docked in the first cavity of the target protein structure. It is very clear from the table given below that the best virtual screened compound binds with higher affinity to the target protein than their best-established compound on the basis of the re-rank score. Apart from rerankscore, we take into consideration the other parameters such as External ligand interactions, protein-ligand interactions, and hydrogen bonds. We see the best virtual screened compound to have lower values for all these parameters than the best established compound, which indicates a better affinity to FLT3 protein structure. Steric values computed by both PLP as well as LJ12-6 methods are again lower for the best virtual screened drug, so is the hydrogen bond value with no directionality.

### Pharmacophore mapping:

**Figure 1:**
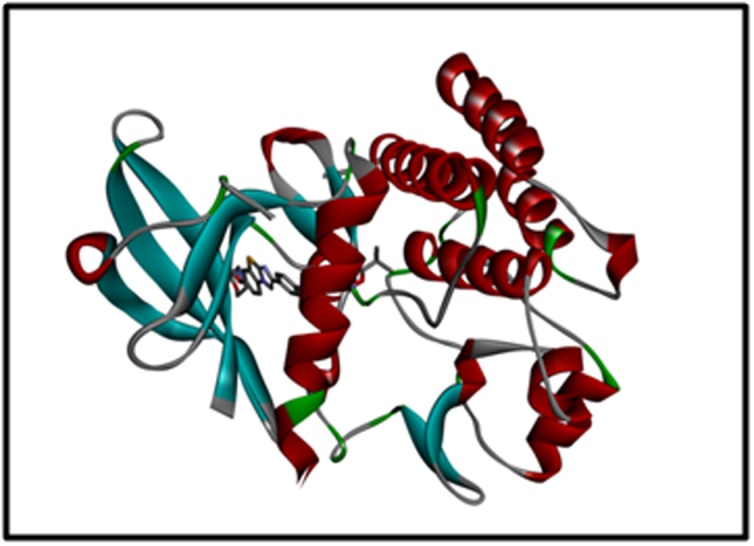
The 3D structure of the FLT3 Protein (PDB ID: 4RT7) visualization in Accelerys Discovery Studio.

Pharmacophore mapping takes into account the molecular features of a ligand for the recognizable arrangement to the target protein. It brings in the optimal spatial systematic topographies of molecular interaction with the target protein receptor. A precise pharmacophore model will help bind a novel structurally diverse ligand to the same receptor site, assisted by annotations and characterized by the aligned poses of the molecule. Figure 1 gives the target protein structure. Binding of the target protein FLT3 with best virtual screened drug PubChem CID 44598530 is realized to be effective and efficient and hence, pharmacophore studies were conducted to improve the understanding of the varying interactions observed in the complex so formed. The interaction studies were carried out only for the purpose of including hydrogen bond interactions, van der Walls interaction, electrostatic interactions, aromatic interactions, and Ligand-binding Interactions.

**Figure 2:**
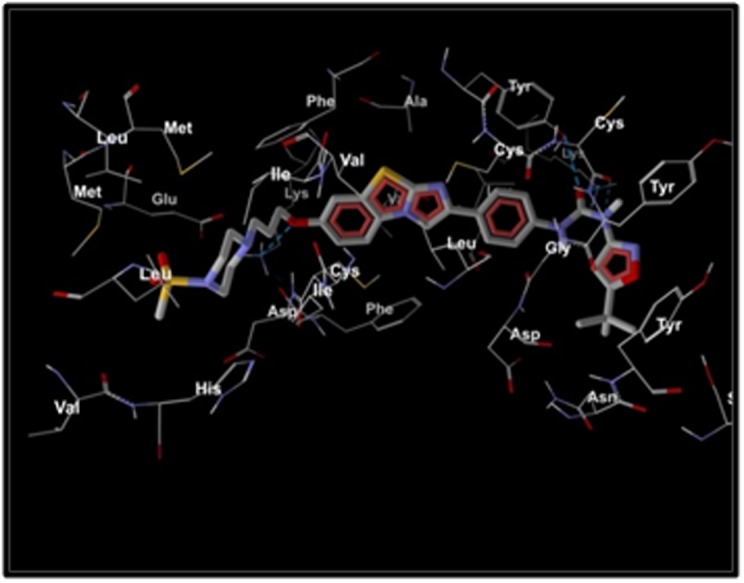
The most effective virtual screened compound (PubChem ID: 44598530) shows Hydrogen Bond interactions

**Figure 3:**
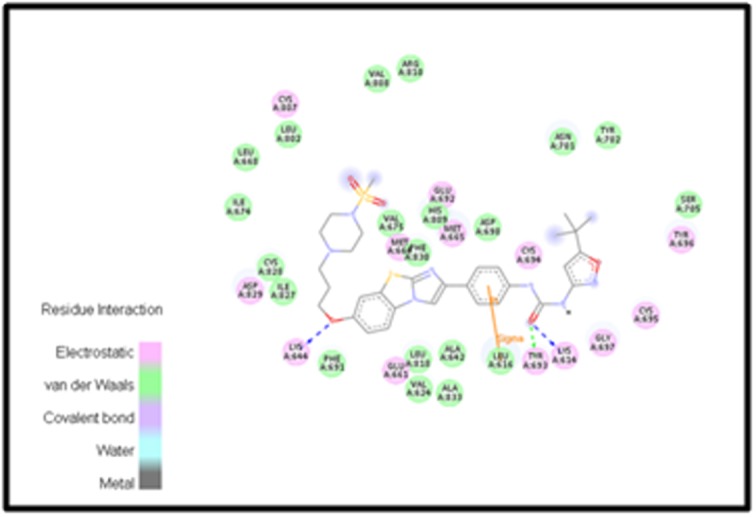
The most effective virtual screened compound (PubChem ID: 44598530) shows van der Walls interaction

**Figure 4:**
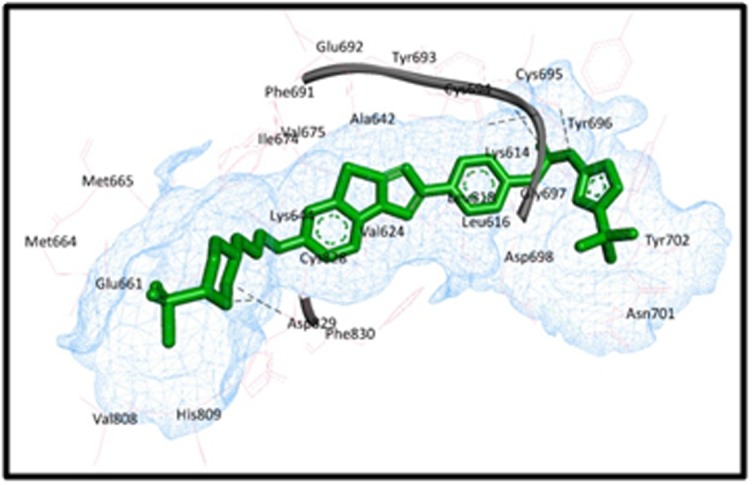
Receptor - Ligand binding between PubChem CID: 44598530 with FLT3 structure.

Figure 2 portrays hydrogen bond interactions of the best virtual screened compound PubChem CID: 44598530 in the cavity of the protein structure of FLT3, highlighting high-affinity H-bond interactions of the compound in the first cavity of the target protein. The dotted lines in light green color show hydrogen bond interaction of the described amino acid present in the receptor from which a stable complex structure arises. The figure presents five Hbond forming amino acids given as Cys 695, Tyr 693, Lys 614, Lys 644 and Cys 828 that bonds with the inhibitor. Figure 3 presents the interacting residues of FLT3 protein structure with compound PubChem CID: 44598530 embedded in its cavity. The residues represented by pink circles exhibit electrostatic interactions whereas those in green exhibit van der Waals (vdW) interactions. Green, as well as blue dotted arrows between the interacting species, denotes hydrogen bonds. Hence, Tyr 693, Lys 614, Lys 644 act as hydrogen bond acceptors and the other 2 hydrogen interactions act as hydrogen bond donors. Also, there is a formation of a sigma- pi bond between the inhibitor and Phe 105. Leu 616 forms a sigma bond with the drug and shows a direct van der Waals (vdW) interaction with the drug. Figure 4 shows the receptor - ligand binding between PubChem CID: 44598530 with FLT3 structure. Binding interactions are indicated by black dotted lines, clearly visible in the figure between the drug and Cys 695, Tyr 693 and Asp 829 in the protein cavity.

### ADMET Profile:

Table 5 provides a comparative account of ADMET property prediction of the best- established compound Quizartinib with PubChem CID: 24889392 and best virtual screened compound having PubChem CID: 44598530. Looking at individual properties it is seen that both the compounds display a positive Blood-Brain Barrier, with the virtual screened drug encasing a lower probability value compared to the established drug. Human Intestinal Absorption (HIA), which provides the prediction of absorption of the compound in the intestine, shows us that the established drug only has a slightly higher increment probability of 0.9953 than the virtual screened drug probability of 0.9709. This shows us that both the drugs can be used in varying dosages and the probability of absorption of either of them is relatively close.

The P-glycoprotein Substrate and P-glycoprotein Inhibitor predictions of both the compounds highlight higher probability values associated with the virtual screened drug when compared to the values associated with the established drug. A difference is observed in the probability of the drug distributed over its centers and its sub-cellular localization in the mitochondria. They have a common distribution center over which the established drug has a 0.5208 probability and the virtual screened drug has a 0.3781 probability. Metabolism predictions varies in points like CYP450 2C9 Substrate, CYP450 2D6 Substrate, CYP450 3A4 Substrate, CYP450 1A2 Inhibitor, CYP450 2D6 Inhibitor, CYP450 2C19 Inhibitor, CYP450 3A4 Inhibitor, CYP Inhibitory Promiscuity with both the compounds acting as non- substrates as well as noninhibitors in all the cases, except in case of CYP450 3A4 Substrate were the established drug and the virtual screened drug is predicted to act as a substrate. CYP450 2C9 Inhibitor is another exception in which the established drug acts as an inhibitor and the virtual screened drug acts like a non-inhibitor.

Toxicity studies infer that both the compounds are non-carcinogens and are not readily biodegradable although the virtual screened drug shows a better result as per the table in these two categories. Both the compounds are also non-AMES toxic but the toxicity value of the established drug is relatively same when compared to that of the virtual screened drug. It can be summarized that the best virtual screened compound displays slightly more preferable probabilities when compared to the best- established compound. Table 6 summarizes the regression prediction comparison of ADMET analysis of the two compounds namely the bestestablished drug and the best virtual screened drug. The regression model highlights that the Rat Acute Toxicity level and the Fish Toxicity has a marginal difference in the readings comparison between the established compound and the virtual screened compound, as given in the table as per the LD50 and pLC50 values respectively. Tetrahymena Pyriformis Toxicity in the virtual screened compound has a lower toxic endpoint level than the established compound.

### Comparative ADMET profile study of the compounds and the control

**Figure 5:**
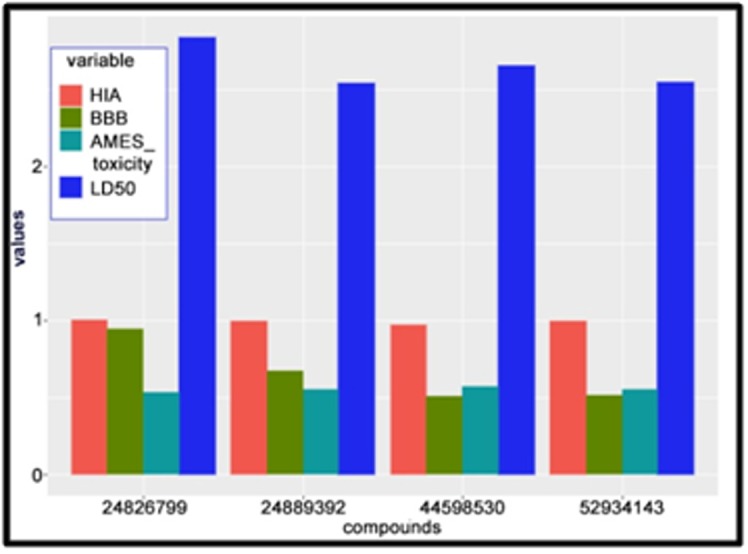
Comparative ADMET studies of BBB, HIA, AMES toxicity and LD50 of the Established compounds against Virtual screened compounds.

A relative ADMET profile comparison was carried out for selected inhibitors by taking the predicted probability values of parameters such as Blood-Brain Barrier (BBB), Human Intestinal Absorption (HIA), AMES Toxicity, and LD50 rat toxicity (Table 7). The two best established compound results from docking were considered of PubChem CID: 124889392 and PubChem CID: 24826799 respectively against the two best virtual screened compound having PubChem CID: 44598530 and PubChem CID: 52934143 respectively. These four compounds were utilized for this study and graphically represented using R-programming as shown in Figure 5. The parameters, BBB, HIA, AMES Toxicity, and LD50 acquired from the admetSAR database were used to tabulate the comparative results according to their estimated values. The best virtual screened compound displays the lowest probability for Blood-Brain Barrier (BBB) and nearby values for AMES toxicity, HIA and LD50 values of all the compounds. The overall profile study is more favorable for the virtual screened compound than the established compound.

### Boiled-egg Plot

**Figure 6:**
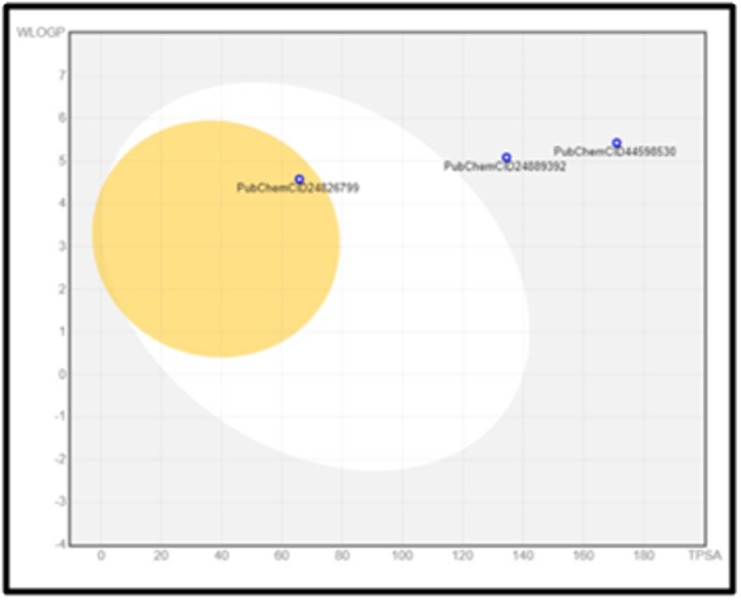
Predictive Model Brain Or IntestinaL EstimateD permeation method (Boiled-egg)

The Boiled-egg model delivers a rapid, easily producible yet robust method to predict the passive gastrointestinal absorption and brain access of small molecules useful for drug discovery and development. The results of the Boiled-egg plot graphically represent the three out of four compounds in (Figure 6). Four drugs were analyzed for the Boiled-egg plot analysis. Two pre-established drugs having PubChemCID: 24889392 and PubChem CID: 24826799 were considered along with two virtual screened drugs having PubChem CID: 44598530 and PubChem CID: 52934143. The selected drugs are tabulated below in Table 8. The result is conclusive of the outside grey region stands for molecules with properties implying predicted low absorption and limited brain penetration which holds good for our best virtual screened drug. It is favorable to the virtual screened drug as it's not Blood-Brain barrier permeant (yellow region). One of the molecules is out of range as its TPSA and WlogP values exceed beyond the graph and hence it’s not shown.

## Conclusion

The best inhibition effect given from the set of established inhibitors was by Quizartinib, which has PubChem CID: 24889392. This compound was tallied and searched against the database to obtain an entirely new ligand, which is the best virtual screened product, with PubChem CID: 44598530. A drugdrug comparative study yielded lower values for External ligand interactions, protein-ligand interactions, hydrogen bonds and rerank score parameters than the established compound pertaining to the higher affinity of the compound to the protein. The stable overall arrangement of the virtual screened ligand-protein complex is done by taking into account all of their features and structure to give us a good result of five H-bond forming amino acids. The positive conclusive result from the boiled-egg plot shows that it does not cross the blood-brain barrier (BBB) and hence, is effective for treating acute myeloid leukemia. This virtual screened compound is better than the other drugs.

## Conflict of Interest

The authors declare no conflict of interest, financial or otherwise.

## Figures and Tables

**Table 1:** List of established FLT3 inhibitors used for the Molecular Docking studies

S. No.	Pub ID	Inhibitor	M.W( g/mol )	HBD	HBA	LogP	References
1	25151352	Pexidartinib	417.82	2	7	4.5	[5]
2	3038522	Tandutinib	562.715	1	8	4.6	[8]
3	49803313	Gilteritinib	552.724	3	10	3.5	[8]
4	5329102	Sunitinib	398.482	3	4	2.6	[9]
5	10366136	Crenolanib	443.55	1	6	3.7	[10][11]
6	126565	Lestauratinib	439.471	3	4	2.2	[10][11]
7	5311	Vorinostat	264.325	3	3	1.9	[11]
8	92136143	TTT-3002	465.513	3	4	1.9	[11]
9	24771867	Alisertib	518.929	2	9	5	[11]
10	3121	Valproic Acid	144.214	1	2	2.8	[11]
11	4261	Entinostat	376.416	3	5	2	[11]
12	25183872	Ixazomib	361.026	4	4	-	[11]
13	65015	Plerixafor	502.796	6	8	0	[11]
14	3062316	Dastanib	488.007	3	9	3.6	[11]
15	91865076	BL-8040	2159.549	34	28	-2.9	[11]
16	23624255	Ganetespib	364.405	3	4	2.3	[11]
17	644241	Nilotinib	529.527	2	9	4.9	[11]
18	25166913	Glasdegib	374.448	3	4	2.4	[11]
19	124518204	PRI-724	548.643	2	4	4.1	[11]
20	89699486	AG-120	582.968	1	9	3.4	[11]
21	89683805	Enasidenib	473.383	3	14	3.5	[11]
22	6253	Cytarabine	243.219	4	5	-2.1	[12][13]
23	24826799	Ponatinib	532.571	1	8	4.1	[12]
24	30323	Daunorubicin	527.526	5	11	1.8	[12]
25	11427553	KW-2449	332.407	2	3	2.7	[12]
26	24889392	Quizartinib	560.673	2	8	5.6	[12]
27	387447	Bortezomib	384.243	4	6	-	[13]
28	42890	Idarubicin	497.5	5	10	1.9	[13][14]
29	285033	Omacetaxine	545.629	2	10	0.8	[15]
30	216239	Sorafenib	464.829	3	7	4.1	[16]
31	49846579	Venetoclax	868.447	3	11	8.2	[17]
32	9829523	Midostaurin	570.649	1	4	4.8	[17]
33	657237	Fludarabine	285.235	4	9	-0.6	[18]
34	6918837	Panobinostat	349.434	4	4	3	[19]
35	451668	Decitabine	228.208	3	4	-1.2	[20][21][22]
36	9444	Azacitidine	244.207	4	5	-2.2	[23]
37	44608567	Epacadostat	438.232	5	11	0.7	[24]
38	16720766	Pevonedistat	443.522	3	8	1.7	[25][26]
39	9933475	Cediranib	450.514	1	7	4.9	[27][28]
40	46216796	Pacritinib	472.589	1	7	3.8	[29]
41	25102847	Carbozantinib	501.514	2	7	5.4	[30]
42	9809715	Nintedanib	539.636	2	7	3.3	[31]
43	20279	Cladribine	285.688	3	7	0.8	[32][33]
44	4212	Mitoxantrone	444.488	8	10	1	[34]
45	216326	Lenalidomide	259.265	2	4	-0.5	[35]
46	36462	Etoposide	588.562	3	13	0.6	[36]

**Table 2:** Established drug docking result

Ligand	Filename	MolDock Score	Rerank Score	HBond	MW
24889392	[00]24889392	-208.882	-172.382	-4.27184	560.667
24826799	[00]24826799	-199.517	-166.743	-3.43569	532.559
24889392	[01]24889392	-201.113	-163.069	-3.88295	560.667
BPR1J-097	[00]BPR1J-097	-196.191	-162.473	-2.5	518.63
24889392	[02]24889392	-194.866	-157.484	-4.17168	560.667
24889392	[03]24889392	-193.507	-150.899	-7.59856	560.667
24826799	[04]24826799	-177.956	-149.896	-1.35889	532.559
24826799	[02]24826799	-185.664	-146.555	0	532.559
3038522	[00]3038522	-178.033	-144.344	-2.41868	562.703
BPR1J-097	[04]BPR1J-097	-174.264	-143.492	-3.81085	518.63

**Table 3:** Virtual screening result

Ligand	Filename	MolDock Score	Rerank Score	HBond	Heavy Atoms	MW
44598530	[00] 44598530	-233.986	-190.091	-5.93428	45	651.799
52934143	[00] 52934143	-205.935	-186.24	-3.81898	47	686.6
1.18E+08	[00]117985880	-230.385	-181.919	-5.51423	44	616.73
66593046	[00] 66593046	-213.982	-177.432	-6.13186	40	560.667
46214474	[00] 46214474	-212.578	-176.9	-7.09361	41	574.651
44598530	[01] 44598530	-216.486	-176.43	-7.29356	45	651.799
24889562	[00] 24889562	-212.896	-175.403	-5.08371	40	560.667
24889562	[01] 24889562	-211.74	-175.349	-6.27097	40	560.667
66592858	[00] 66592858	-219.171	-175.152	-5.06128	40	560.667
66798938	[00] 66798938	-209.913	-175.032	-10.5716	41	574.694

**Table 4:** Drug-drug comparative study result

Established Drug	Virtual Screened Drug
Energy overview: Descriptors	MolDock Score	Rerank Score	MolDock Score	Re-rank Score
Total Energy	-209.504	-172.881	-230.32	-187.558
External Ligand interactions	-230.614	-199.032	-251.6	-217.967
Protein - Ligand interactions	-230.614	-199.032	-251.6	-217.967
Steric (by PLP)	-225.723	-154.846	-245.115	-168.149
Steric (by LJ12-6)		-40.313		-44.681
Hydrogen bonds	-4.891	-3.874	-6.485	-5.136
Internal Ligand interactions	21.109	26.152	21.28	30.409
Torsional strain	8.82	8.273	13.933	13.069
Torsional strain (sp2-sp2)		2.713		3.379
Hydrogen bonds		0		0
Steric (by PLP)	12.672	2.18	7.723	1.328
Steric (by LJ12-6)		12.986		12.632

**Table 5:** ADMET Predicted Profile (Classification data)

Established CID:	24889392	24889392	Virtual Screened CID:	44598530
Model Absorption	Result	Probability	Probability	Result	Probability
Blood-Brain Barrier	BBB+	0.6667	0.6667	BBB+	0.5054
Human Intestinal Absorption	HIA+	0.9953	0.9953	HIA+	0.9709
Caco-2 Permeability	Caco2-	0.6097	0.6097	Caco2-	0.5795
P-glycoprotein Substrate	Substrate	0.8191	0.8191	Substrate	0.8336
P-glycoprotein Inhibitor	Inhibitor	0.6701	0.6701	Inhibitor	0.7209
	Non-inhibitor	0.5257	0.5257	Inhibitor	0.6238
Renal Organic Cation Transporter	Non-inhibitor	0.7678	0.7678	Non-inhibitor	0.698
Distribution					
Subcellular localization	Mitochondria	0.5108	0.5108	Mitochondria	0.3781
Metabolism					
CYP450 2C9 Substrate	Non-substrate	0.7672	0.7672	Non-substrate	0.647
CYP450 2D6 Substrate	Non-substrate	0.7552	0.7552	Non-substrate	0.7158
CYP450 3A4 Substrate	Substrate	0.7028	0.7028	Substrate	0.6679
CYP450 1A2 Inhibitor	Non-inhibitor	0.7846	0.7846	Non-inhibitor	0.7466
CYP450 2C9 Inhibitor	Inhibitor	0.5216	0.5216	Non-inhibitor	0.5854
CYP450 2D6 Inhibitor	Non-inhibitor	0.8896	0.8896	Non-inhibitor	0.8742
CYP450 2C19 Inhibitor	Non-inhibitor	0.5837	0.5837	Non-inhibitor	0.6634
CYP450 3A4 Inhibitor	Inhibitor	0.6802	0.6802	Inhibitor	0.5338
CYP Inhibitory Promiscuity	High CYP Inhibitory Promiscuity	0.6105	0.6105	High CYP Inhibitory Promiscuity	0.5361
Toxicity					
Human Ether-a-go-go-Related Gene Inhibition	Weak inhibitor	0.7897	0.7897	Weak inhibitor	0.7319
	Inhibitor	0.7464	0.7464	Inhibitor	0.8014
AMES Toxicity	Non AMES toxic	0.5521	0.5521	Non AMES toxic	0.5673
Carcinogens	Non-carcinogens	0.7503	0.7503	Non-carcinogens	0.6483
Fish Toxicity	High FHMT	0.9946	0.9946	High FHMT	0.9917
Tetrahymena Pyriformis Toxicity	High TPT	0.9585	0.9585	High TPT	0.9316
Honey Bee Toxicity	Low HBT	0.6523	0.6523	Low HBT	0.6905
Biodegradation	Not ready biodegradable	1	1	Not ready biodegradable	0.9937
Acute Oral Toxicity	III	0.6144	0.6144	III	0.5688
Carcinogenicity (Three-class)Non-required	0.5287	0.5287	Non-required	0.5686

**Table 6:** ADMET Predicted Profile (Regression analysis)

Established CID	Virtual Screened CID:
Model Absorption	Value	Unit	Value	Unit
Aqueous solubility	-3.8824	LogS	-3.7914	LogS
Caco-2 Permeability	0.6625	LogPapp, cm/s	0.5066	LogPapp, cm/s
Toxicity				
Rat Acute Toxicity	2.5432	LD50, mol/kg	2.6542	LD50, mol/kg
Fish Toxicity	1.2335	pLC50, mg/L	1.3616	pLC50, mg/L
Tetrahymena Pyriformis Toxicity	0.5649	pIGC50, ug/L	0.547	pIGC50, ug/L

**Table 7:** Comparative ADMET profile of the test ligands and the control is given (ec1, ec2 – established drugs, vs1, vs2 – virtual screened drug, all in the ascending order).

Compound	HIA	BBB	AMES toxicity	LD50
24889392(ec1)	0.9953	0.6667	0.5521	2.5432
24826799 (ec2)	1	0.9444	0.5331	2.8377
44598530 (vs1)	0.9709	0.5054	0.5673	2.6542
52934143(vs2)	0.9926	0.5117	0.5468	2.5484

**Table 8:** Boiled egg plot comparison values

Molecule	MW	TPSA	XLOGP3	MLOGP	GI absorption	BBB permeant
PubID 24889392	560.67	134.4	5.64	3	Low	No
PubID 24826799	532.56	65.77	4.11	3.9	High	Yes
PubID 44598530	651.8	170.93	5.43	2.54	Low	No
PubID 52934143	686.6	134.4	8.25	3.66	Low	No
